# Iron, Ferritin, Hereditary Ferritinopathy, and Neurodegeneration

**DOI:** 10.3389/fnins.2019.01195

**Published:** 2019-12-11

**Authors:** Barry B. Muhoberac, Ruben Vidal

**Affiliations:** ^1^Department of Chemistry and Chemical Biology, Indiana University-Purdue University Indianapolis, Indianapolis, IN, United States; ^2^Department of Pathology and Laboratory Medicine, Indiana Alzheimer Disease Center, Stark Neurosciences Research Institute, Indiana University School of Medicine, Indianapolis, IN, United States

**Keywords:** hereditary ferritinopathy, neurodegeneration, mutant ferritin, ROS, ferritinophagy, ferroptosis

## Abstract

Cellular growth, function, and protection require proper iron management, and ferritin plays a crucial role as the major iron sequestration and storage protein. Ferritin is a 24 subunit spherical shell protein composed of both light (FTL) and heavy chain (FTH1) subunits, possessing complimentary iron-handling functions and forming three-fold and four-fold pores. Iron uptake through the three-fold pores is well-defined, but the unloading process somewhat less and generally focuses on lysosomal ferritin degradation although it may have an additional, energetically efficient pore mechanism. Hereditary Ferritinopathy (HF) or neuroferritinopathy is an autosomal dominant neurodegenerative disease caused by mutations in the FTL C-terminal sequence, which in turn cause disorder and unraveling at the four-fold pores allowing iron leakage and enhanced formation of toxic, improperly coordinated iron (ICI). Histopathologically, HF is characterized by iron deposition and formation of ferritin inclusion bodies (IBs) as the cells overexpress ferritin in an attempt to address iron accumulation while lacking the ability to clear ferritin and its aggregates. Overexpression and IB formation tax cells materially and energetically, i.e., their synthesis and disposal systems, and may hinder cellular transport and other spatially dependent functions. ICI causes cellular damage to proteins and lipids through reactive oxygen species (ROS) formation because of high levels of brain oxygen, reductants and metabolism, taxing cellular repair. Iron can cause protein aggregation both indirectly by ROS-induced protein modification and destabilization, and directly as with mutant ferritin through C-terminal bridging. Iron release and ferritin degradation are also linked to cellular misfunction through ferritinophagy, which can release sufficient iron to initiate the unique programmed cell death process ferroptosis causing ROS formation and lipid peroxidation. But IB buildup suggests suppressed ferritinophagy, with elevated iron from four-fold pore leakage together with ROS damage and stress leading to a long-term ferroptotic-like state in HF. Several of these processes have parallels in cell line and mouse models. This review addresses the roles of ferritin structure and function within the above-mentioned framework, as they relate to HF and associated disorders characterized by abnormal iron accumulation, protein aggregation, oxidative damage, and the resulting contributions to cumulative cellular stress and death.

## Introduction

Life has evolved incorporating iron as a crucial element in a number of metabolic processes by utilizing it in biochemical catalysis, electron transfer, and oxygen transport and storage. Familiar are the hemeproteins hemoglobin and myoglobin which perform the latter two processes, cytochrome-c for electron transport, and the cytochromes P-450 which perform drug metabolism. In addition, there are major metabolic pathways that require non-heme iron enzymes as centers of catalytic transformation. Neurotransmitter synthesis is intimately connected with such enzymes, e.g., tryptophan hydroxylase and tyrosine hydroxylase, which perform the initial reactions transforming tryptophan and tyrosine into neurotransmitters. These examples imply a clear requirement that cellular iron be adequately available and properly compartmentalized for normal physiology. However, the processes of acquisition, distribution, and catalytic utilization of iron in the cellular environment, which can be rich in oxygen and reducing equivalents, is not without risk (Muhoberac and Vidal, 2013). Iron is a redox active transition metal, which makes it substantially different from sodium, potassium, calcium, and zinc in that it is often involved in single electron transfers. Improperly coordinated iron in the labile iron pool (LIP) of cells can produce reactive oxygen species (ROS) that modify the structure of proteins, lipids, and nucleic acids, causing an immediate local problem of loss of normal function, but also protein aggregation through protein destabilization and unfolding. Additionally, proteins with mutations alone are often destabilized enhancing susceptibility to unfolding and aggregation. Finally, iron also has the ability to enhance aggregation of proteins that have regions of intrinsic sequence disorder, which are generally considered as normal functional sequences that are independent of mutation or ROS modification, and some of these aggregation-sensitive proteins are key players in several neurodegenerative diseases. For example with Parkinson disease (PD), alpha-synuclein, which has as its normal sequence a partially disordered stretch of amino acids that take up variable structural conformations, is especially sensitive to iron levels in that iron enhances its aggregation (Wolozin and Golts, 2002; Theillet et al., 2016). Similarly, the partially disordered amyloid β (Aβ) peptide in Alzheimer disease (AD) undergoes aggregation with iron (Liu et al., 2018). Furthermore, these aggregates have the ability to induce iron redox changes as well as generate ROS (Hands et al., 2011; Liu et al., 2018). Both dysregulation of iron metabolism and iron accumulation in specific regions of the brain are directly associated with several less common neurodegenerative diseases, i.e., the Neurodegeneration with brain iron accumulation (NBIA) diseases (Rouault, 2013; Meyer et al., 2015), but also apparently with several other more commonly encountered ones (Oshiro et al., 2011). To address the issues of availability of iron as metabolically crucial and the potential toxicity of improperly coordinated iron, ferritin has evolved as the end point protein to remove and store excess iron safely and provide it to the cell as required, and thus to reduce cellular damage and stress.

The importance of native ferritin in normal physiology, as well as its purified availability for *in vitro* research, has made it one of the more well-studied proteins over several decades (Crichton, 2009). While the mechanism of iron uptake and storage as an iron mineral in its interior is complex but relatively well-understood, the mechanism of iron release, although generally considered to involve lysosomal degradation through the process of ferritinophagy, has research suggesting alternative pathways. These alternatives are release (1) induced by small cytosolic molecules usually found close to ferritin or (2) by the proteasome (Liu et al., 2003; DeDomenico et al., 2009; Tang et al., 2018). Such pathways may be involved in general or perhaps more nuanced iron management. More recently, mutant forms of ferritin in which the C-terminal alpha helix is disordered and unraveled at the four-fold pores providing an iron exit and entry pathway that is normally considered closed, have been characterized (Muhoberac and Vidal, 2013). These mutant forms were discovered through clinical investigation and molecular-level characterization of the neurological disorder hereditary ferritinopathy (HF) or neuroferritinopathy, which has some clinical characteristics similar to PD. Inclusion bodies (IBs) containing ferritin, increased iron levels, and oxidative damage (carbonylation) are found in brain samples of patients with HF upon autopsy (Vidal et al., 2004). These characteristics are to a great extent reproducible for investigation with cellular and animal models expressing mutant ferritin. Such ferritin expressed and purified from cell cultures undergoes both (1) precipitation with increasing iron and (2) oxidative damage, i.e., carbonylation, proteolysis, and crosslinking, in the presence of physiological concentrations of iron and ascorbate found in the brain (Baraibar et al., 2012). Here ascorbate functions as a reductant so that iron can produce ROS. *Dealing with elevated iron, ROS formation, aggregation, oxidative damage, and IB formation in HF, requires the use of cellular synthesis, transport, repair, and disposal mechanisms and may provide a source of cumulative cellular stress and damage as cells age*.

The finding of elevated iron in HF, PD, AD, and other neurodegenerative diseases suggests the importance of tight physiological control of iron compartmentalization and concentrations, and leads to consideration of ferroptosis, which is a newly described form of rapid programmed cell death clearly different in cellular structural changes and biochemical pathways from apoptosis and necrosis (Dixon et al., 2012; Stockwell et al., 2017). Characteristics of ferroptosis are lipid peroxidation and abundant or elevated iron. Ferroptosis may be enhanced through the process of ferritinophagy, by which ferritin is delivered to the lysosome for degradation and a large quantity of iron can be rapidly released enhancing ROS production (Latunde-Dada, 2017). However, the overall ferroptotic process is complicated in that damage can be repaired by a particular glutathione peroxidase if sufficient glutathione is available. Importantly, ferroptosis appears to be strongly connected with cell death in neurodegenerative diseases (Tang et al., 2018). In HF there are elevated iron and lipid peroxidation similar to ferroptosis, but at least three mechanisms of ferritinophagy inhibition may be operative, suggesting that it is the leaky four-fold pores and modestly elevated iron that triggers a long term cellular ferroptotic-like state gradually and cumulatively leading to cellular misfunction and decline. A contribution to decline is the accumulation of ferritin aggregates from ferritin overproduction and iron binding, which causes a variety of stressful secondary effects on synthesis, transport, and disposal, as well as providing additional ROS generating sites. The complicated interrelationships between the molecular-level processes described above and HF are reviewed herein, with the further objective that this presentation will provide a better understanding of the common threads among protein aggregation, elevated iron, and ROS damage found in neurodegenerative diseases.

## Genetics, Clinical Presentation, and Pathology of HF

HF or neuroferritinopathy is inherited in an autosomal dominant pattern. Linkage analysis established a relationship between the disease and a locus on chromosome 19q13.3, which contains the ferritin light chain (*FTL*) gene, consisting of four exons and three introns (Curtis et al., 2001). Mutations in the *FTL* gene causing HF have been reported in individuals with a Caucasian ancestry and in East Asian populations from Japan, Korea, and China, presenting with abnormal involuntary movements (Curtis et al., 2001; Vidal et al., 2004; Mancuso et al., 2005; Ohta et al., 2008; Devos et al., 2009; Kubota et al., 2009; Storti et al., 2013; Nishida et al., 2014; Ni et al., 2016; Yoon et al., 2019). Mutations in *FTL* consist of nucleotide duplications in exon 4 that affect the C-terminal residues of the FTL polypeptide (Table 1). There are no known polymorphisms in the *FTL* gene that may affect the clinical and pathological phenotype. In addition to the cases indicated in Table 1, two more cases of HF have been described. One case was diagnosed pathologically and no genetic data is available (Schröder, 2005). The second case consists of a missense mutation (A96T) in the *FTL* gene in an individual without significant involvement of the putamen, thalamus, and substantia nigra that did not show autosomal dominant transmission since the mother of the proband, also a carrier of the A96T mutation, had similar MRI findings and was asymptomatic (Maciel et al., 2005). The A96T variant has been recently shown to be stable under physiological conditions and incorporate iron comparable to that of wild-type FTL ferritin (Kuwata et al., 2019).

**Table 1:** *FTL* genetic variants associated with Hereditary Ferritinopathy (neuroferritinopathy).

**Mutation**	**Amino acid change**	**References**
c.439_440 het_dupG	p.Asp147GlyfsX34	Yoon et al., 2019
c.442dupC	p.His148ProfsX33	Mancuso et al., 2005
c.439_442dupGACC	p.His148ArgfsX34	Kubota et al., 2009
c.458dupA	p.His153GlnfsX28	Devos et al., 2009
c.460dupA	p.Arg154LysfsX27	Curtis et al., 2001
c.467_470dupGTGG	p.Gly157GlyfsX24	Ni et al., 2016
c.468_483dup16	p.Leu162TrpfsX24	Nishida et al., 2014
c.469_484dup16	p.Leu162ArgfsX24	Ohta et al., 2008
		Storti et al., 2013
c.497_498dupTC	p.Phe167SerfsX26	Vidal et al., 2004

The onset of clinical signs and symptoms may occur between the second and seventh decade of life and depending on the specific mutation, the disease may become evident from the third to the fifth decade of life (Vidal and Ghetti, 2015). HF occurs equally in males and females, with duration ranging from a few years to several decades (Curtis et al., 2001; Vidal et al., 2004, 2011; Mancuso et al., 2005; Ohta et al., 2008; Devos et al., 2009; Kubota et al., 2009; Ory-Magne et al., 2009; Storti et al., 2013; Nishida et al., 2014; Ni et al., 2016; Yoon et al., 2019). The clinical presentation of the disease may be highly variable not only between families but also within a family, suggesting that a heterogeneous presentation may be a characteristic of HF (Ory-Magne et al., 2009). The disease may present as a movement disorder with tremor, cerebellar signs, Parkinsonism, dystonic, and choreic movement; in addition, pyramidal and pseudo-bulbar symptoms may be present. Although dystonia and dysarthria are the main manifestations of HF, the disease cannot be diagnosed only on the basis of clinical symptoms alone. In more advance stages, psychiatric and cognitive symptoms may become evident (Curtis et al., 2001; Vidal et al., 2004, 2011; Mancuso et al., 2005; Chinnery et al., 2007; Ohta et al., 2008; Devos et al., 2009; Kubota et al., 2009; Ory-Magne et al., 2009; Storti et al., 2013; Nishida et al., 2014; Ni et al., 2016)

Neuroimaging studies allow regional characterization of normal vs. abnormal iron deposition through T2^*^-weighted images (WI) and susceptibility weighted imaging (SWI). These studies show in the early clinical stage of HF the presence of an abnormal decrease in T2^*^ signal intensity reflected as hypo-intense lesions in the basal ganglia on particularly in the globus pallidus and putamen, which signifies abnormal accumulation of iron (Curtis et al., 2001; Vidal et al., 2004, 2011; Mancuso et al., 2005; Chinnery et al., 2007; McNeill et al., 2008; Ohta et al., 2008; Devos et al., 2009; Kubota et al., 2009; Ory-Magne et al., 2009; Ohta and Takiyama, 2012; Storti et al., 2013; Nishida et al., 2014; Ni et al., 2016; Yoon et al., 2019). With the progression of the disease the signal loss extends to the dentate nucleus, substantia nigra, and cerebral cortex.

**Figure 1:**
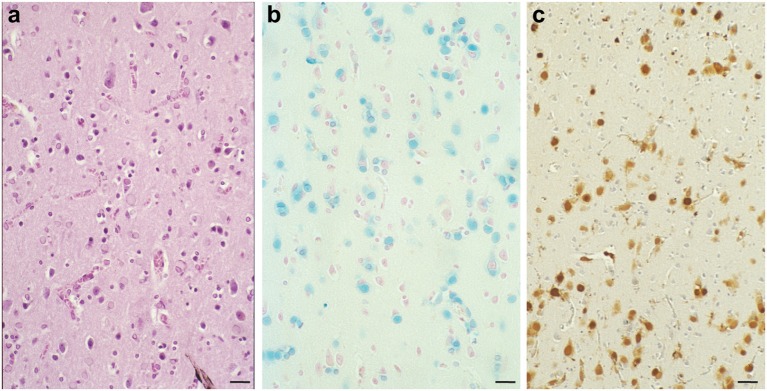
Numerous inclusion bodies containing iron are present in the brain with a case of hereditary ferritinopathy. In HF, the most striking pathologic alteration is the presence of intranuclear and intracytoplasmic bodies in glial cells and in some subsets of neurons. Sections of the putamen show numerous ferritin bodies of various sizes stained by H&E **(a)**. Iron-containing ferritin inclusion bodies stained with the Perls' method for iron **(b)** and immunostained using antibodies specific for the mutant ferritin light chain polypeptide **(c)**. FTL immunoreactivity is seen in the nuclei and also in the cytoplasm. Perls' staining and antibodies against wild-type and mutant FTL subunits were used as previously described (Vidal et al., 2004). Scale bars: **(a–c)**, 50 μm.

**Figure 2:**
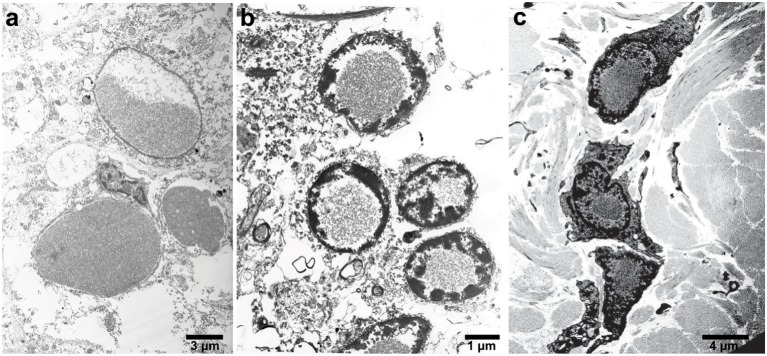
Electron microscopy of IBs in patients with HF. High-power electron micrograph showing ferritin accumulation in the putamen **(a)**, in the nucleus of granule cells of the cerebellum **(b)**, and skin **(c)**. Note the chromatin accumulation toward the nuclear membrane.

Macroscopic examination of the brain of patients with HF reveals mild cerebral and cerebellar atrophy as well as cavitation of the putamen. Neuropathological data is available for individuals with the c.442dupC, c.460dupA, and c.497_498dupTC mutations (Curtis et al., 2001; Vidal et al., 2004; Mancuso et al., 2005). The caudate nucleus and putamen may show a grayish discoloration. At neuropathologic examination, the main findings are the presence of ferritin IBs in the cytoplasm and nuclei of glial cells and some subsets of neurons, and abnormal iron deposition in IBs, in particular in the caudate nucleus, putamen, and globus pallidus. IBs can be seen as eosinophilic, homogenous bodies on haemotoxylin and eosin staining, and can be stained using the Perls' and Turnbull blue methods for iron (Figure 1; Vidal et al., 2004). Different from other IBs such as those containing α-synuclein in Lewy bodies in PD and dementia with Lewy bodies (DLB) (Spillantini and Goedert, 2018), ferritin IBs do not show electron dense fibrillar structures on electron microscopy (Figure 2) and cannot be stained by thioflavin S, but are strongly immunopositive using antibodies against ubiquitin. In the cytosol, ferritin may be dispersed or forming IBs showing a spherical appearance. Intranuclear IBs measure between 2 and 35 μm in diameter, and may occupy almost completely the nucleus and as a result displace the chromatin up against the nuclear membrane. Antibodies against the N-terminus of FTL as well as antibodies against ferritin heavy chain (FTH1) and mutant-specific antibodies strongly label ferritin IBs of all sizes in glial cells and in nerve cells (Vidal et al., 2004). The use of antibodies also show the presence of diffuse deposits, perhaps early aggregates, in the cytoplasm (Figure 1; Vidal et al., 2004. Markers for lipid oxidation are also evident by immunostaining (Mancuso et al., 2005).

In the neocortex, intranuclear and intracytoplasmic ferritin immunopositivity is present throughout the cortical layers, with the exception of layer I and II (Vidal and Ghetti, 2015). Intranuclear IBs are seen in perineuronal satellite cells and in perivascular glia. The caudate nucleus, putamen, and globus pallidus contain the largest numbers of IBs. In these areas, extracellular ferritin aggregates may also be found, probably as the result from the coalescence of multiple bodies. Intranuclear IBs may be seen in neurons of the putamen, globus pallidus, and thalamus, and occasionally, ferritin immunopositivity may be seen in the leptomeningeal and parenchymal vessel walls as well as in leptomeningeal cells. In the cerebellum, IBs are seen in the nuclei of glial cells, including the Golgi epithelial cells, in granule cells and Purkinje cells. In the latter, IBs could also be found in the cytoplasm of the perikaryon and dendrites. Glial cells of the white matter are affected as well. IBs are also found in the nuclei of endothelial cells, in cells of the vascular adventitia and in the epithelium of the choroid plexuses; ependymal cells appear to be free of IBs. IBs have been reported in the skin, kidney, liver, and muscle in affected individuals from French and American families (Vidal et al., 2004; Mancuso et al., 2005). The detection of ferritin IBs in biopsies of the skin or muscle may be helpful for the pathologic diagnosis of symptomatic individuals (Figure 2; Vidal et al., 2004, 2011).

Low serum ferritin levels were initially reported in individuals with the c.460InsA mutation (Curtis et al., 2001); however, serum ferritin levels are decreased in some but not all patients with HF (Muhoberac and Vidal, 2013; Yoon et al., 2019). Interestingly, CSF ferritin levels were significantly lower than normal in a patient with the c.469_484dup16 mutation who had normal serum ferritin values (Nishida et al., 2014). Additional studies are needed to establish whether CSF ferritin levels could be used as a novel biomarker for HF.

## Modeling HF in Cells and Mice

**Figure 3:**
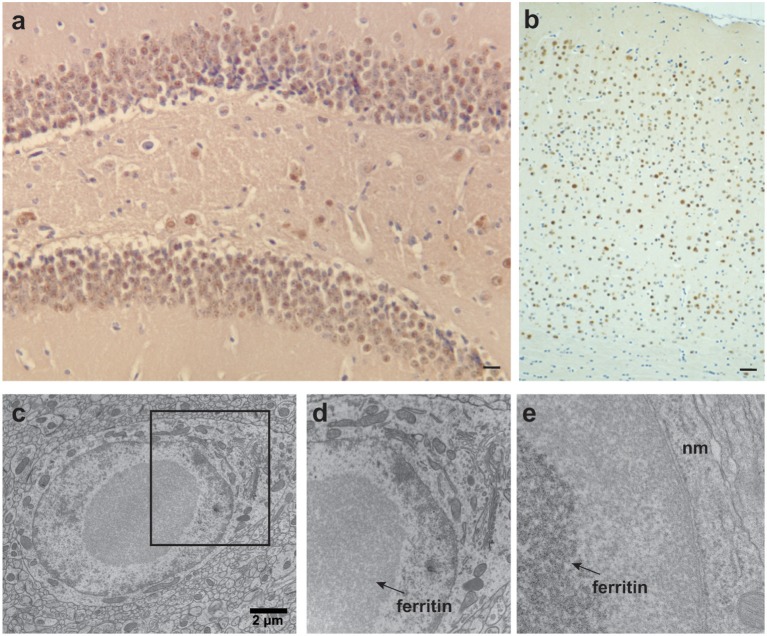
Ferritin inclusions in FTL-Tg mice. Histological and immunohistochemical studies of a 343-days-old FTL-Tg heterozygous mouse. **(a)** Ferritin inclusions are present in all areas of the hippocampus, in particular in the granular layer of the dentate gyrus and CA4. **(b)** Intranuclear and intracytoplasmic ferritin deposits in the cerebral cortex immunolabeled by antibodies against the FTL polypeptide. **(c–e)** Ultrastructural studies showing a ferritin inclusion body in the nucleus of a neuron of the striatum **(c)**. The section marked in **(c)** was magnified in **(d,e)**. IBs (indicated with arrows) occupy most of the nucleoplasm. The chromatin appears to be centrifugally displaced to varying degrees, often forming a thin layer adjacent to the nuclear membrane (nm) **(e)**. **(a,b)** Immunohistochemistry using antibodies against wild-type FTL subunits as previously described (Vidal et al., 2008). Scale bars: **(a)**, 10 μm; **(b)**, 40 μm; **(c)**, 2 μm.

A mouse model of HF (FTL-Tg) that expresses the human mutant ferritin FTL subunit p.Phe167SerfsX26 (mtFTL) has been extensively characterized and also used to generate astrocytes and primary mouse embryonic fibroblasts for *in vitro* studies (Barbeito et al., 2009; Li et al., 2015) that complemented studies using fibroblasts from patients with HF (Barbeito et al., 2010). Expression of the transgene in the mouse yields a progressive neurological phenotype, with a significant decrease in motor performance, shorter life span, misregulation of iron metabolism, and evidence of oxidative damage (Vidal et al., 2008; Barbeito et al., 2009; Deng et al., 2010). Mutant HF mice form nuclear and cytoplasmic ferritin IBs in glia and neurons throughout the CNS and in organ systems outside the CNS, as found in patients with HF (Figure 3; Vidal et al., 2004, 2008). Both cytosolic and nuclear inclusions grow larger in size and number as the animals age to the point of crowding other cellular structures potentially causing mechanical disruption of cellular processes like transport. DNA oxidative damage is detected associated with nuclear mitochondria (Deng et al., 2010) at 12 months but not 6 months of age. Ferric iron accumulation is detected throughout the CNS through Perls' Prussian blue staining in histological sections and T2-weighted imaging *in vivo*. There is evidence of proteosomal involvement at the ferritin inclusions, including ubiquitination and the presence of the 11S and 20S subunits. Ferritin inclusions from the transgenic mice are SDS insoluble as found in HF patients. The SDS insoluble IBs suggests a rather strong association of the ferritin 24-mers (Vidal et al., 2008), which could occur through iron bridging as might be expected with a disordered and extended C-terminus and perhaps including contributions from partial unfolding and proteolysis. Additional studies show a substantial increase in cytoplasmic ferritin (~200–400%) depending on the subunit identity from antibody labeling and cortex vs. cerebellum sampling (Barbeito et al., 2009). There is a statistically significant increase in non-heme iron levels between controls and transgenic HF mice of ~15% (cortex) to 20% (cerebellum), but these levels are more modest than the protein increases above. Evidence is found for elevated oxidative protein and phospholipid damage through detection of HNE-protein modifications, protein carbonyls, and lipid peroxidation products like MDA in the transgenic HF mice. Radical formation is found through immunohistochemistry of nitrone-protein adducts. *Thus, this mouse model produces only modest increases in iron levels vs. controls, but substantial, cumulative overproduction and lack of clearance of ferritin, as well as oxidative damage*.

Similar dysfunction is observed in primary culture skin fibroblasts with the FTL subunit p.Phe167SerfsX26 mutation obtained from a HF patient (Barbeito et al., 2010). The HF fibroblasts show accumulation of mutant ferritin in the cytoplasm and nucleus. There is a substantial increase in ferritin content in the HF cells of ~320 and 470% in the FTH1 and FTL subunit concentrations, respectively, but the increase in total iron content is again modest at 25%. Also, a modest increase in ROS concentration is found in the HF cells over normal, which was further enhanced by FAC treatment.

In primary mouse embryonic fibroblasts (MEFs) from FTL-Tg mice, there is initially a substantial increase in the levels of soluble mtFTL, but not in the levels of insoluble mtFTL. After incubation with ferric ammonium citrate (FAC), MEF cells became less viable (3–5 days) and contained more iron than MEF cells from wild-type mice by ~40%. Thus, the combination of mutant-containing ferritin and iron leads to aggregation into SDS resistant aggregates (Garringer et al., 2016). Incubation with the iron chelator deferiprone (DFP) after iron loading removes substantially more iron from the cells than the controls, which suggests the iron is more loosely bound in the cells expressing mtFTL as would be expected if the iron is initiating aggregation and relatively loosely bound to the C-termini and surface region of ferritin spheres rather than internally as a mineral. In earlier studies, transgenic astrocytes were loaded with iron by FAC incubation causing substantial conversion of ferritin to a SDS insoluble form, but incubation with the iron chelator phenanthroline led to a substantial reversal back to a soluble form (Baraibar et al., 2008). Along these lines, *in vitro* studies using purified mtFTL homopolymeric recombinant ferritin that was precipitated by iron can be partially resolubilized by addition of the chelator deferoxamine (Baraibar et al., 2008).

**Figure 4:**
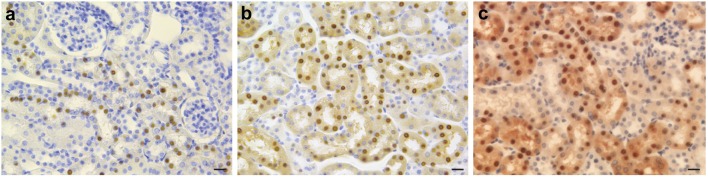
Immunohistochemistry of kidney in iron-loaded and control FTL-Tg mice. Sections of the kidney of control **(a)**, iron treated **(b)**, and DFP treated **(c)** FTL-Tg mice immunostained using antibodies specific for the mutant ferritin light chain polypeptide as previously described (Garringer et al., 2016). Iron loaded mice appear to have a larger number of IBs while DFP treated FTL-Tg mice showed the presence of a diffuse ferritin staining in tubule cells of the kidney. Scale bars: **(a–c)**, 20 μm.

An interesting but perhaps counterintuitive result is obtained in studies of FTL-Tg mice under iron loading and chelation treatment (Garringer et al., 2016). Here, iron overload and DFP treatment of the mouse model have remarkable effects on systemic iron homeostasis and ferritin deposition, without significantly affecting CNS pathology (Figure 4). The data obtained using the FTL-Tg mouse model is in agreement with previous observations indicating lack of effectivity of a chelation therapy in individuals affected by HF (Chinnery et al., 2007; Kubota et al., 2009; Storti et al., 2013). Chinnery et al. (2007) treated three patients with monthly venesection for 6 months. Two of the patients also were treated with intravenous deferoxamine (4,000 mg weekly subcutaneously for up to 14 months), and one had oral DFP (2 g, three times a day for 2 months). These treatments cause profound and refractory iron depletion without significant benefits for the patients. Kubota et al. (2009) treated a patient with monthly venesections (400 mL/mo) for 2 months without any change in the clinical condition of the patient. Storti et al. (2013) did a 6-month trial with DFP (15 mg/kg/day) in one patient without any signs of improvement. Work on the mouse model also suggests that systemic ferritin deposits in HF (Vidal et al., 2004) may not be useful to monitor therapeutic approaches since they may be modified independently from brain ferritin deposits.

To determine whether loss of function of the FTL subunit in the brain could lead to some of the pathologic features observed in HF by loss of the iron storage function of ferritin, a *Ftl* knock-out (*Ftl*^−/−^) mouse model was generated (Li et al., 2015). Interestingly, Ftl^−/−^ mice are viable (knock-out of the *Fth* subunit is embryonic lethal) and do not show signs of neurodegeneration, presence of an inflammatory process, noticeable protein aggregates, or iron accumulation as in patients with HF, suggesting that the deleterious effect(s) caused by mutant FTL subunits in HF are driven by disruption of the ferritin pore structure and unraveling of the C-terminus in the heteropolymer rather than by a loss of normal function of the FTL subunit itself. Importantly, *Ftl*^−/−^ mice show that Fth ferritin homopolymers are capable of maintaining brain iron homeostasis *in vivo*, paving the way for the development of a potential therapeutic approach for HF using RNA interference to induce sequence-specific post-transcriptional gene silencing of mutant *FTL* (Li et al., 2015).

## Ferritin Structure, Function, and Normal Iron Incorporation

The functions of ferritin are to (1) sequester and store excess ferrous iron, when necessary in large amounts, removing it from the LIP and thus preventing deleterious cellular processes such as ROS generation, protein aggregation, and iron deposition, and (2) to allow release of this iron as needed for cellular processes. Storage is accomplished by converting ferrous iron into a less reactive and compact form of ferric iron oxide “mineral” that is stored in the interior of ferritin, which has the form of a spherical shell to contain it. Spatial storage efficiency (i.e., density of the iron mineral) is high in that storage does not require separate iron ions be chelated by a single protein or a small molecule chelator in a 1:1 or 1:2 ratio.

**Figure 5:**
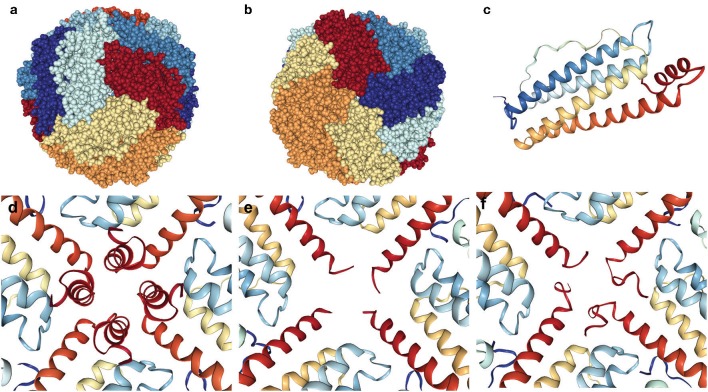
Ferritin 24-mer spherical shell emphasizing the disordered four-fold pore in HF. The three-fold and four-fold pores of the ferritin shell are found at the center of the spheres **(a,b)**. The ferritin subunits are colored differently for emphasis, and a single subunit **(c)** is pictured from above (outside) the shell with the short red E-helix pointing inwards. The intact four-fold pore **(d)** contrasts two examples **(e,f)** of the many different C-terminal conformations from unraveling and disorder revealed by X-ray crystallography of the p.Phe167SerfsX26 mutant FTL. Pictures are made from crystallographic structures in the RCSB database using files 2fg8, 2ffx, and 4v6b (mtFTL), and **(d–f)** are interior views.

Ferritin is a molecule assembled from 24 protein subunits into a highly symmetric protein shell (Figures 5a,b) of exterior diameter ~12 nm and a hollow core of ~8 nm that can store up to 4,500 iron atoms within its shell (Harrison and Arosio, 1996; Crichton and Declercq, 2010). Native human ferritin is a heteropolymer composed of FTH1 (MW~21 kDa) and FTL (MW~20 kDa) subunits in varying ratios depending on the cell/organ of origin such that its storage rate and ability is optimized for its location. Both subunits are composed of four parallel and closely associated alpha-helix bundles (A–D) and a fifth alpha-helix (E) that is shorter and not aligned with the others but rotated approximately perpendicular (~60 degrees; Figure 5c). The E helix points inwards from the ferritin spherical surface and forms the sides of a pore (see below). The two different subunits diverge in amino acid identity (~55%), but fold into an almost identical 3-dimensional structure, which allows them to assemble into a highly symmetric shell with tight fitting inter-subunit surfaces and junctions exhibiting three-fold and four-fold symmetry (Figures 5a,b). This assembly forms eight more polar three-fold pores of ~6 Å length and ~3.4 Å diameter and six more apolar four-fold pores of <3 Å diameter and ~12 Å length, with the latter generally considered closed to iron passage (Harrison and Arosio, 1996; Crichton and Declercq, 2010). The FTH1 subunit contains the ferroxidase center, which oxidizes the ferrous ions in preparation for mineral formation and the FTL subunit contains clusters of negatively charged residues which foster the nucleation and mineral growth. The three-fold pores are the ferrous iron entry pathways and there is a group of negatively charged residues that help transport the iron through the pore, along the interior of the shell to the ferroxidase site, and then to the nucleation and mineralization points. The specifics of the iron acquisition, transport, catalytic oxidation, and deposition processes in ferritin, i.e., identification of the specific metal binding amino acid residues involved in the ferroxidation and the overall pathway from ferrous iron to ferric mineral, have been elucidated in detail over a decade or more of study (Masuda et al., 2010; Tosha et al., 2010; Behera and Theil, 2014; Pozzi et al., 2015). Interestingly, homopolymeric ferritin 24-mers composed of only FTL subunits can load iron into the shell, but at a much reduced rate vs. heteropolymeric ferritins, which can coordinate the iron deposition process between subunits.

## Mutant Ferritin Structural and Functional Anomalies

Over the last decade there has been a substantial amount of research describing the molecular level structural and functional differences between normal ferritins and those containing mutant FTL subunits made possible by cloning, bacterial expression, and purification. Our studies focused on the p.Phe167SerfsX26 mutation that causes a 9 amino acid substitution and a 16 amino acid extension of the C-terminus out of a total 174 amino acids (Baraibar et al., 2010). This substantial change into a completely non-native sequence does not prevent mtFTL subunits from assembling into homopolymeric 24-mer spheres, with ultrastructure quite similar to the native, that are crystallizable. This mutation encompasses part of the E helix, but as demonstrated by X-ray crystallography in different subunits of a 24-mer the helix unraveling can be found to range from just before the mutation to 10 or more amino acids toward the D helix. The unraveling distribution (i.e., the number of amino acids disordered as to not be visible by X-ray crystallography) is statistical over the six four-fold pores and the four C-termini composing each of them (Figures 5d–f). The result is a substantial disordering of what was a tight four-fold pore into a more flexible, iron permeable structural defect and an unraveling of the C-terminal sequence that, if extended from the exterior of the shell, could reach as far as the entire diameter of ferritin. This statistical disordered structure is important not just for iron leakage, but because the disordered C-termini will take up various orientations and conformations as potential iron binding sites. Several amino acid residues of the disordered C-terminus are known iron binding ligands and the disorder and conformational sampling adds variability to their potential iron chelation geometry in a search for free energy minimization. As mentioned previously, redox active improperly coordinated iron is problematic as a ROS generator causing protein (including ferritin) modification, and the multiple C-termini conformations may enhance the generation probability. Additionally, sampling multiple conformations can enhance the probability of iron bridging two C-termini either on a single mutant-containing ferritin or between two mutant-containing ferritins enhancing aggregation (Muhoberac and Vidal, 2013). Indeed, because of the extremely close packing of the cytoplasm, this C-terminal extension has the potential to interact with multiple cellular constituents, e.g., small molecules, proteins, bilayers, cytoskeleton component, etc., with unpredictable results.

*In vitro* studies show that the four-fold pore-centered FTL mutation has several consequences. Firstly, homopolymeric ferritin composed of mtFTL subunits is more susceptible to iron-loading induced aggregation and precipitation than ferritin composed of wild type FTL (wtFTL) subunits. In standard iron-loading assays, precipitation occurs starting at approximately an iron to mtFTL ferritin (24-mer) ratio of 1,500:1, with homopolymers of wtFTL remaining soluble to >4,000:1 ratio (Baraibar et al., 2008). Although the iron concentration was high, these experiments point out fundamental differences in the interaction with and handling of iron between mutant and wild-type ferritins. Secondly, with heteropolymeric ferritin, this aggregation tendency extends to the cases in which mtFTL subunits are combined with those of either wtFTL or FTH1 to form ferritin 24-mers (Muhoberac et al., 2011). Specifically, reduction in the percent of mtFTL subunits by half and having the remainder provided by either wtFTL or by FTH1 causes no aggregation at an iron to ferritin ratio of 1:1,000 (as above) but substantial aggregation at a 3,000:1 ratio. Clearly, protection is not provided by a large reduction in the number of mutant subunits. Thirdly, the overall stability of homopolymeric ferritin composed of mtFTL subunits is compromised with respect to that of the wtFTL in temperature denaturation and proteolysis studies (Baraibar et al., 2008). However, it is known that wtFTL homopolymers are more stable than FTH1 homopolymers such that the inclusion of wtFTL subunits in native heteropolymeric ferritin is considered stabilizing. Although the loss of stability of the homopolymeric mtFTL 24-mers is found to not be substantial, any loss suggests the possibility of more general enhanced local unfolding causing repositioning and exposure, if only transiently, of normally buried or hydrogen bonded residues. Supporting this unfolding process is the enhanced thermolysin proteolysis of mtFTL over wtFTL homopolymers in which a 17 kDa N-terminal fragment is produced from the mutant but not the wild type. The position of this fragment in the sequence strongly suggests substantial transient fluctuations in overall mutant ferritin structure even though ultrastructurally the mutant is spherical and crystallizable. Fourthly, susceptibility to ROS damage of mtFTL homopolymers is enhanced substantially over that of ferritin composed exclusively of wtFTL subunits. At physiological concentrations of iron and ascorbate, mtFTL homopolymers undergo substantial proteolysis (6 and 14 kDa fragments), crosslinking (27 kDa fragment), and carbonylation (Baraibar et al., 2012). Ferritin composed of wtFTL subunits alone does not undergo these changes and a hydroxyl radical scavenger prevents mtFTL homopolymer from undergoing such disruptive modifications implying a mutant ferritin-centered, ROS-generating reaction initiated by iron and ascorbate. These results strongly suggest that the mtFTL homopolymer has one or likely more iron binding sites available that generate ROS which attack ferritin and may attack other proteins and lipids, as discussed in the next section.

With respect to the function of iron incorporation, defects are found in both the mtFTL homopolymer and the 24-mer heteropolymers. A comparison of iron uptake between heteropolymers composed of half FTH1 and half of either mtFTL or wtFTL subunits demonstrates a 50% reduction in iron incorporation with a 1,000:1 ratio of iron to heteropolymeric ferritin (Muhoberac et al., 2011). This ratio is below that found to cause iron precipitation suggesting there exists an iron incorporation defect caused by the four-fold structural perturbation in HF that exists at the level of single ferritin heteropolymers containing the mutant subunit. In addition, small soluble aggregates not yet large enough to form precipitates could also hinder the sequestration of iron. Taken together, the above-mentioned *in vitro* studies suggest that the overall structural and functional effects of the mutation are quite substantial and damaging, and may require a variety of cellular processes for correction and compensation, which are energetically costly to the cells. It is interesting to note that in PD there is a decrease in the concentration of FTL polypeptides in the substantia nigra, which in a manner parallels the mtFTL dysfunction in HF in that both lead to similar changes in LIP iron (Koziorowski et al., 2007; Friedman et al., 2011).

## Iron Chemistry, Interactions, and Reactive Oxygen Species

The importance of iron to cellular function must be understood within the context of its versatile, but complex chemical behavior and its difficulty in cellular processing, transport, and storage (Shriver and Atkins, 2006; Crichton and Declercq, 2010). Iron can not only undergo redox cycling between ferrous and ferric states, but its ability to be cycled at a particular redox potential strongly depends on its cellular ligation, i.e., the ligands from proteins or small molecules that are bound to it. Ferrous iron prefers coordination to nitrogen or sulfur-containing ligands whereas ferric iron prefers those that contain oxygen such as aspartate and citrate. This preference is part of a chemical classification of binding that is dependent on atomic radius and polarizability of the atoms in contact (Shriver and Atkins, 2006). Iron coordination not only varies its redox potential, but can determine its behavior as an electron relay or a catalytic center, and such processes generally require some level of conformational control of as well as outright exchange of iron coordination by the chelating protein and substrate. Reductant concentration and cellular packing also factor into the iron redox state. Iron can interact with dioxygen producing potentially toxic ROS that modify proteins and phospholipids, and this reaction is substantially enhanced by iron with <6 coordinate ligand shell (Graf et al., 1984). Such improperly coordinated iron is found, even transiently, in the LIP with small cytosolic molecules and with mutant, denatured, and aggregated proteins. Even protein aggregation and precipitation of unmodified proteins, especially those with regions of intrinsic sequence disorder, can be enhanced substantially by iron presence, as is found with α-synuclein and Aβ (Mantyh et al., 1993; Golts et al., 2002; Everett et al., 2014; Boopathi and Kolandaivel, 2016). Indeed, there are iron deposits and interactions with specific proteins that are routinely found in association with neurodegenerative diseases (Zecca et al., 2004; Carboni and Lingor, 2015; Ward et al., 2015; van Bergen et al., 2016; Liu et al., 2018). Aqueous iron solubility itself varies substantially between its ferrous and ferric forms, with the ferric form basically insoluble near neutral pH and requiring cellular carriers such as small molecule chelators or proteins, but with ferrous iron soluble near 100 mM (Crichton and Declercq, 2010). Because of this level of molecular behavioral complexity and the number of cellular constituents with which iron can interact, the cell has developed a variety of mechanisms to employ iron in biochemical catalytic processes as well as to deal with excess iron and iron-produced ROS, which potentially negatively effects proteins, lipids, and DNA.

The most common cellular ROS—hydrogen peroxide, superoxide, and the hydroxyl radical—vary substantially in their origin, interactions, lifetime, and toxicity (Halliwell and Gutteridge, 2007; Auten and Davis, 2009; El-Beltagi and Mohamed, 2013; Davies, 2016). The hydroxyl radical is considered the most toxic with a high reactivity and short diffusion distance, causing protein backbone cleavage, oxidative residue modification, crosslinking, nucleic acid modification, and lipid peroxidation close to its sight of generation, although once initiated the peroxidation damage can spread. Hydrogen peroxide is not particularly reactive with a large ability to diffuse and is part of a redox signaling mechanism in cells, and superoxide is considered of intermediate toxicity. There are enzymes that normally produce these ROS for biochemical and defensive purposes, and ROS may also originate with damaged enzymes or those that on occasion miscycle during normal catalysis (Turrens, 2003). The characterization of ROS availability is further complicated by the potential interconvertability of these species both by detoxifying enzymes and local concentrations of poorly coordinated iron in the LIP.

Hydroxyl radical formation is generally discussed with respect to two widely accepted and employed chemical generating systems of different composition (Cohen et al., 1981; Uchida et al., 1989; Ito et al., 1993). The first consisting of hydrogen peroxide and a ferrous salt is the Fenton/Haber-Weiss-type reaction which produces hydroxyl radicals (·OH) as follows:

$$\begin{array}{l} {\text{Fe}}^{2+}\;+\;{\text{H}}_{2}O_{2}\;\to \;{\text{Fe}}^{3+}+\;\cdot \text{OH }+\;{\text{OH}}^{-} \end{array}$$

The ferrous ion is regenerated by a reaction between a ferric iron and hydrogen peroxide or perhaps superoxide $\left(\cdot O_{2}^{-}\right)$ which is formed from ferrous iron and dioxygen, and then the cycle repeats. The second generating system consists of ascorbic acid, EDTA, and ferric iron and is called the Udenfriend-type reaction. This system appears to be more similar to what might occur *in vivo* in that it has a supply of physiological reductant and the iron is chelated by ligands other than water, hydroxide, and/or oxygen. Ascorbic acid reduces the iron, which reacts with dioxygen to produce superoxide. Then superoxide produces hydrogen peroxide which feeds a Fenton/Haber-Weiss-type reaction producing the hydroxyl radical. Ascorbic acid cycles the iron back to the reduced state producing additional hydroxyl radicals. As might be expected, the ability of the Udenfriend-type reaction to generate ROS is strongly dependent on the type of iron chelation in that EDTA-iron must transiently become 5-coordinate opening a ROS generating site. Radical generation and the ability to modify target molecules is strongly modulated by the choice of iron chelators that are substituted for EDTA (Burkitt and Gilbert, 1990).

There are a number of cellular enzymatic processes to (1) eliminate the specific ROS, converting them to water or other ROS that are, under normal conditions in which iron levels are controlled, less damaging, or (2) repair cellular damage caused by them (Halliwell and Gutteridge, 2007; Kim et al., 2015). Superoxide is catalyzed to hydrogen peroxide by superoxide dismutase, thus exchanging one ROS for another, which can generate a potentially more toxic species through the non-enzymatic Fenton/Haber Weiss-type reactions. Hydrogen peroxide can be catalyzed to water by catalase, removing it from the toxicity cycle. Glutathione peroxidases can also convert hydrogen peroxide to water, but doing so requires reduced glutathione, which brings into focus the need for maintaining the reducing potential in the brain where normal levels of ascorbate and reduced glutathione (GSH) are relatively high (1–2 mM) (Baraibar et al., 2012). However, high levels of reducing agents in the brain may be problematic with respect to ROS generation. *Taken together, ROS management and damage repair are highly complex and interrelated processes that would likely become problematic as iron levels exceeded the norm*. In addition, protein conformational changes and aggregation that result in iron binding sites have the potential to further contribute to ROS generation, as will be discussed later.

## Protein and Lipid Modification and Degradation by ROS

The general characteristics of biological exposure to ROS are deleterious oxidative modifications of proteins, lipids, and DNA (Halliwell and Gutteridge, 2007; Suzuki et al., 2010; Yin et al., 2011; El-Beltagi and Mohamed, 2013; Davies, 2016). As with iron, the levels of oxidized cellular proteins and lipids increase with age and are found in HF and other neurodegenerative diseases. Additionally, modified proteins and their aggregates are associated with compromised proteasomal function inhibiting damaged protein clearance (Grune et al., 2001; Stadtman, 2004; Hipkiss, 2006; Radak et al., 2011). Thus, this lack of clearance and increased concentration not only leads to enhanced protein aggregation in a cyclical manner, but strains cellular repair and elimination processes to the point of accumulation, diverting cellular resources from other tasks (e.g., biomembrane maintenance). Although ROS-centered cellular damage is often characterized by protein carbonyl derivative formation (carbonylation), the kinds of modifications that are caused by ROS are much more varied (Halliwell and Gutteridge, 2007). Protein side chains can undergo substantial modification by the hydroxyl radical depending on their identity. For example, lysine and arginine can acquire a carbonyl, methionine can be oxidized, phenylalanine, and tyrosine can each acquire an added hydroxyl group, and histidine becomes 2-oxo-histidine. Such modifications change residue physical attributes, e.g., charge and size, and also chemical reactivity, which in turn can alter their interactions with substrates or other proteins, decrease protein stability, and enhance aggregation without direct iron binding or bridging. Protein modification and unfolding has the potential to enhance iron binding and iron binding-induced aggregation. *Loss of protein stability (partial unfolding) and the aggregation process itself can produce new iron binding sites that can form centers of ROS generation*. Potentially more extensive protein damage can occur because the hydroxyl radical can also cause polypeptide backbone cleavage and crosslinking, with the latter particularly problematic for protein repair and disposal pathways.

Not only proteins, but lipids are subject to modification by ROS (Yin et al., 2011; El-Beltagi and Mohamed, 2013), with those containing more than one double bond being particularly susceptible to radical attack and the resulting substantial structural damage (Wagner et al., 1994). Lipid peroxidation has been linked to a large number of diseased states including AD and PD as well as being found with HF (Yin et al., 2011). Such damage can clearly alter biomembrane structure, e.g., fluidity and phospholipid raft formation, and function, including that of passive transport and membrane bound enzymes and receptors. Even phospholipid-based signaling can be affected. *However, the extent of potential cellular disruption and damage is magnified by the more complicated cyclical propagation behavior of ROS-modified lipids that attack unaltered lipids and the several kinds of reactive phospholipid byproducts that are formed, e.g., malondialdehyde (MDA) and 4-hydroxynonenal (HNE) that cause additional damage* (Halliwell and Gutteridge, 2007). There are three steps involved in radical-centered damage to membranes: initiation, propagation, and termination, with various sub-reactions including direct iron involvement (Wagner et al., 1994; El-Beltagi and Mohamed, 2013). Lipid peroxidation most likely begins with an initiation step in which a hydrogen atom is extracted by a hydroxyl radical from a carbon adjacent to double bonded carbons in a polyunsaturated hydrocarbon chain (LH to L·). The ability energetically to produce this carbon-centered radical depends on its location relative to vinyl groups. The hydrogen of a carbon sandwiched between two vinyl groups, i.e., a bis allyic carbon, is most easily abstracted followed by a carbon adjacent to a single vinyl group, i.e., an allylic carbon. The most difficulty hydrogen to abstract is an acyl chain carbon away from double bonds explaining the susceptibility of polyunsaturated lipids to radical attack. In the propagation steps, dioxygen reacts with the carbon-centered radical on the acyl chain (L·) forming a peroxyl radical (LOO·), which has the ability to extract another hydrogen from a second unmodified polyunsaturated hydrocarbon chain producing additional damage through a second carbon-centered radical (L·) and a lipid hydroperoxide (LOOH). This reaction is cyclical in propagating damage. The lipid hydroperoxide is relatively stable but can react with iron forming hydroxyl and alkoxyl radicals (LO·), of which both can abstract hydrogen. Iron involvement highlights increased iron levels and improperly coordinated iron in neurodegenerative diseases. Termination involves the formation of more stable or non-radical products (e.g., crosslinked LL), trapping by small molecule radical scavengers, or enzymatic repair. However, it should be noted that lipid hydroperoxide breakdown into reactive byproducts such as MDA and HNE, which are well-known markers for ROS damage and cause additional lipid and protein damage. For example, both MDA and HNE can cause numerous types of protein modifications, e.g., crosslinking between two proteins and modification of the structure of residues, thus hindering protein function and fostering aggregation. Taken together, iron can both initiate lipid and protein damage by hydroxyl radical formation, as well as enhance the rate of damage in a cyclical manner, especially with lipids. These two reactive byproducts are proximal markers for cell death involving uncontrollable lipid peroxidation through ferroptosis, as will be discussed shortly.

## Ferritin Iron Release by Ferritinophagy and Other Mechanisms

Under circumstances in which cellular iron is lacking, iron stored in native ferritin can be released for cytosolic distribution, and there are different potential mechanisms of various complexity and divergent pathways that may do so. Such mechanisms are lysosomal and proteasomal ferritin degradation, and removal of iron through the ferritin three-fold pores, which have clearly been established as the entrance channels for iron but not as exit pathways. Current focus as the most likely and dominant iron release mechanism in normal cells is lysosomal degradation of ferritin through an autophagic process called ferritinophagy (Biasiotto et al., 2015; Ndayisaba et al., 2019), which is complicated and requires substantial cellular machinery, i.e., transport, synthesis and degradation. However, there is an extensive literature on completely different mechanisms (Crichton, 2009; DeDomenico et al., 2009; Bou-Abdallah et al., 2018), and it is not unreasonable to suggest that a fine tuned cell would have more than one way to remove iron from ferritin under different conditions, especially one that minimizes use of cellular material and energy and is not spatially dispersed. Degradation of ferritin by ferritinophagy is a complex and spatially non-local process requiring significant cellular resources of synthesis and transport, and even proteosomal degradation is energetically costly and requires ATP both for labeling the target proteins with ubiquitin and the degradation process itself. Perhaps cells have also developed a more measured, fine-tuned, iron release pathway utilizing locally available cellular components such as cytosolic reductants and small molecule chelators, and such pathways have undergone substantial *in vitro* investigation (Melman et al., 2013; Koochana et al., 2018). *Thus, there may be two (or more) mechanistic levels of stored iron release from native ferritin operating concurrently in the cell, one for nuanced, local iron balance and the other used under different circumstances, e.g., under iron-depleted cellular distress*. In fact, given the importance of iron management, evolutionary sampling, and the complexity of cellular response to environmental change, it is not unexpected that there would be multiple mechanisms to inhibit and augment iron release from ferritin, which is an ancient protein. Below is discussed ferritinophagy in the context of an autophagic process as well as the alternate more local mechanisms of iron release.

Of the two major protein degradation pathways, ubiquitin-proteasomal and lysosomal degradation, ferritinophagy depends upon the latter through a multistep pathway involving autophagosomes and the cargo protein Nuclear Receptor Coactivator 4 (NCOA4) (Mancias et al., 2014, 2015; Bellelli et al., 2016; Gatica et al., 2018). In this pathway, NCOA4 binds ferritin and is necessary for ferritin to be transported to lysosomes. First, ferritin binds to NCOA4 forming a complex through an FTH1 subunit. Next, the double walled proto-autophagosome membrane encompasses the ferritin NCOA4 complex forming a completely enclosed structure. Finally, the autophagosome fuses with the lysosome in which ferritin is degraded releasing iron. This pathway requires microtubules for transport and assembly (Hasan et al., 2006). Further details of the NCOA4-ferritin interaction are from (1) binding studies of a cloned and expressed portion of the NCOA4 protein (residues 383–522) and (2) its different interactions with FTH1, wtFTL, and single amino acid mutations of the FTH1 subunit (Gryzik et al., 2017). The FTH1 ferritin 24-mer can bind up to 24 NCOA4 proteins. Furthermore, FTH1 binds to the NCOA4 fragment tightly (in the nM range), but NCOA4 does not bind to wtFTL or to FTH1 after certain mutations. This binding specificity may be crucial to the explanation of the accumulation of ferritin into IBs, as will be discussed below.

HF is characterized by formation of IBs that are composed of mtFTL, wtFTL, and FTH1 subunits, and there is clear biochemical and histologic evidence of the accumulation of all three types of ferritin subunits in patients with HF and in the mouse model (Vidal et al., 2004, 2008). However, the ferritinophagy process is based on recognition of FTH1 in the ferritin 24-mer for lysosomal degradation and clearance, and structurally complete 24-mers can be formed from any combination of these subunits. Brain ferritin has FTH1 as the predominant subunit of its normal ferritin 24-mer assembly, and it is possible that imbalance in the ratio of mtFTL and/or wtFTL to FTH1 subunits caused by HF, i.e., a decrease in normal ferritin FTH1 subunit content in the 24-mer, could substantially and negatively affect NCOA4 binding to ferritin and its clearance by ferritinophagy. This could explain, at least in part, the buildup of IBs in HF. More likely, inhibition of NCOA4 binding to ferritin could be caused by just the presence of the mtFTL in the 24-mer because of interference from the extended C-terminus or by formation of ferritin aggregates by iron bridging. *Thus, the buildup of cellular ferritin in the form of IBs is consistent with direct mutant-based inhibition of ferritinophagy through compromised NCOA4-FTH1 subunit binding*. Inhibited ferritinophagy is particularly problematic in HF in that the elevated iron from four-fold pore misfunction is a driver to overproduction of both normal and mutant ferritin without appropriate clearance. This feedback process would likely tax cellular resources and eventually cause ferritin accumulation with multiple unwanted cellular interactions, e.g., association with susceptible proteins, inhibition of microtubule-based cellular transport, and altered spatial packing in a cumulative manner. In addition, mutant-containing ferritin, together with cellular reductants and iron would generate ROS. With ferritinophagy compromised and ferritin accumulation the cell would likely resort to proteasomal degradation of ferritin and the aggregates, and there is evidence of proteasomal constituents in IBs (Vidal et al., 2008). However, the proteasome is ill-equipped to handle ferritin because of its size vs. the dimensions of the proteasome interior. Ferritin 24-mers might dissociate into monomers, which are substantially smaller, however both normal and mutant-containing ferritin are particularly stable. Further complicating disposal is that protein aggregates inhibit the proteasome. Partitioning of the proteasome into the vicinity of the ferritin aggregates may be a failed attempt to remove ferritin or it may be in response to ROS damage of other proteins caused by ferritin-iron aggregates. *Taken together ferritin overexpression and compromised ferritinophagy are major components of HF, and mutant-induced inhibition of binding of ferritin to NCOA4 may contribute directly to small aggregate and IB formation*.

Other proposed mechanisms of inhibition of ferritinophagy are dependent on elevated iron. More specifically, in the absence of HF, high iron levels (1) enhance proteosomal degradation of NCOA4 and (2) inhibit the binding between FTH1 subunit and NCOA4 (Mancias et al., 2015; Gryzik et al., 2017). However, the modest elevation of iron in HF suggests that neither process may dominantly inhibit ferritinophagy, and the mutation-based direct inhibition of may contribute in parallel with, or perhaps even dominate, the other two mechanisms. In addition, the mechanism by which ferritinophagy is decreased by iron-induced enhancement of proteosomal degradation of NCOA4 may not be operative in HF with the proteasome compromised by ferritin aggregates and unable to remove NCOA4. In any case, there may be as many as three mechanisms working to inhibit ferritinophagy at some stage of the progression of HF consistent with the gradual buildup of IBs.

Several alternative native ferritin iron release mechanisms that do not require complex cellular responses, but are instead more direct pathways centered on ferritin interaction with locally available cytosolic effector molecules, have been proposed from *in vitro* studies (Boyer et al., 1988; Crichton, 2009; Melman et al., 2013; Badu-Boateng et al., 2017; Johnson et al., 2017; Koochana et al., 2018). Most of the latter do however require (1) reduction of the iron mineral stored in the interior of the ferritin, (2) an iron chelator, and (3) a pathway through which the chelator and iron-chelator complex can pass through the shell. Cytosolic molecular reductants, e.g., NADPH and FADH, and chelators are relatively large bringing into question their ability to pass through the three-fold pores and gain access to the ferric iron mineral, as well as the rates in which these processes could occur. By X-ray crystallography the pores are smaller than the dimensions of these cytosolic effector molecules. Even if the reductant does not need to enter the ferritin interior because electrons could transit the shell by a redox relay mechanism, the standard three-fold pore configuration is not designed for chelated ferrous iron to have egress. Still, there have been reports of slow release of iron from the ferritin interior by treatment with combinations of reductants and chelators, and there is evidence that the crystallographic, nominal three-fold pore diameter of ~3.4 Å relaxes to accommodate somewhat larger diameter molecules (Yang and Nagayama, 1995). In any event, such iron release would likely be slow compared with lysosomal iron release. In HF, limitations in reductant and chelator entry and exit are substantially reduced with the disordered four-fold pore.

Another iron release mechanism involves perturbing ferritin three-fold pore structure directly. Such studies were performed through amino acid mutations at the pore, binding of peptides to the surface of ferritin, and exposure to low levels of structural perturbants with human FTH1 24-mer ferritin and a frog FTH1 analog (Takagi et al., 1998; Jin et al., 2001; Liu et al., 2003, 2007; Hasan et al., 2008). Site-directed mutagenesis of specific amino acids e.g., FTH1 L138P, allows substantial modulation of the iron egress rate by increasing pore size and/or disorder. Furthermore, small peptides that bind to the surface of ferritin, which were found through screening assays, and even low concentrations of physiological perturbants modulate the iron exit rate of native frog ferritin. Thus, it is plausible that one or more pathways alternate to ferritinophagy may be operative in the overall process of cellular iron balance, although available reducing ability is necessary to dissolve the mineral core in all cases. These processes have the potential for more nuanced control of iron levels, perhaps with more rapid response and energetic efficiency than ferritinophagy. However, deconvoluting these competitive processes in a cellular environment without one masking another and the often use of excesses of perturbants, reductants, and chelators further complicates the already complicated scheme of iron balance.

## Cellular Stress and Destruction Through Ferroptosis

Ferroptosis is becoming increasingly characterized as a major player in several neurodegenerative diseases (including AD and PD) as well as being understood to be a new form of Programmed Cell Death differing biochemically and morphologically from apoptosis, classical autophagy, and necrosis (Biasiotto et al., 2015; Gao et al., 2016; Angeli et al., 2017; Stockwell et al., 2017). Furthermore, close sequential biochemical linkage between the end point of ferritinophagy (iron release) and the ROS damage in ferroptosis suggests that ferritinophagy iron release is a component of, or perhaps an outright driver of, ferroptosis under certain conditions (Santana-Codina and Mancias, 2018; Sui et al., 2018, 2019). Interestingly, components of the ferroptotic process have been reported in the literature as isolated effects for some time now, but their connections into an overall picture were lacking. This was in part because cellular studies from diverse cell types found at different times that iron chelators, antioxidants (radical scavengers), and lipoxygenase inhibitors can cause inhibition of ferroptotic cell death (Cao and Dixon, 2016; Stockwell et al., 2017). Conversly, a different set of drugs and small molecules were found to induce ferroptosis. Thus, it has been argued that ferroptosis is not really new and there is a suggested timeline to the evolution of its etiology (Hirschhorn and Stockwell, 2019). *The two major cellular components characterizing ferroptosis are (1) iron presence or elevation and (2) accumulated lipid damage with inhibition of the lipid repair process*. The pathways of several contributors to the overall ferroptotic process can be deconvoluted experimentally and recognized individually using small molecule ferroptosis inducers and inhibitors specific to each contributor (Cao and Dixon, 2016; Stockwell et al., 2017), although not all have been applied to cells involved in neurodegeneration. These studies are complicated in that ferroptosis is often induced by one small molecule and inhibited by another.

Paralleling HF, a picture of the crucial importance of iron availability or excess in ferroptosis is clear, and under some circumstances its excess may induce ferroptosis (Fang et al., 2018; Dixon and Stockwell, 2019). Rapid ferroptosis-induced cell death can be inhibited by preventing normal activity of the NCOA4 cargo protein and its ability to bring ferritin to the lysosomes for degradation through ferritinophagy, which releases toxic levels of iron into the LIP (Mancias et al., 2014; Gao et al., 2016). Iron chelators are able to circumvent ferroptosis initiated by several different small molecule inducers that work through different cellular pathways, but contribute to the overall ferroptotic process (Cao and Dixon, 2016). Furthermore, changing expression of ferroportin and transferrin receptor levels, i.e., exporters and importers of cellular iron, modulate ferroptosis as expected (Gao et al., 2016; Geng et al., 2018; Dixon and Stockwell, 2019). Recently, it was demonstrated that intracellular iron overload caused by treatment with a membrane permeable iron chelation can cause ferroptosis and cell death (Fang et al., 2018). Such studies implicate, in addition to the need for iron availability, that an increase in iron level itself may be a mechanism of ferroptosis initiation, especially in stressed cells where other compensating processes are depressed.

Lipid peroxidation is a defining process of ferroptosis leading to cell death, and there are two major pathways which can cause this: (1) ROS generated at improperly coordinated iron non-specifically attacking surrounding lipids and proteins, as well as iron enhancing the rate of lipid autooxidation, and (2) an iron-containing lipoxygenase (LOX) which detrimentally modifies phospholipids in a more specific (enzymatic) manner and requires dioxygen but not reducing equivalents. Both pathways likely occur to some extent in the ferroptotic process and the dominant one would depend on the amount of LIP iron accumulation without safe storage vs. LOX availability and expression. Recently, it was found that LOX inhibitors may more effectively function as radical scavengers than inhibitors (Shah et al., 2018), thus adding weight to improperly coordinated iron and ROS as a drivers of ferroptosis. Importantly, the process of ferroptosis-induced cell death is inhibited by phospholipid repair by a specific glutathione peroxidase (GPX4), but requires GSH to do so, and it is known that direct covalent inhibition of GPX4 prevents repair of damaged phospholipids enhancing ferroptosis (Yang and Stockwell, 2016). Restriction of GSH availability by inhibition of GSH synthesis or prevention of import of its precursor cystine also enhances ferroptosis.

In HF, the situation is more complicated in that (1) sequestration of iron into ferritin is already compromised to some extent with the leaky four-fold pores causing chronically elevated iron, which enhances ROS damage without the need for ferritinophagy releasing iron, and (2) the true extent of ferritinophagy inhibition in HF is unclear but may be substantial because there is significant but gradual ferritin IB accumulation. Moderately elevated iron level and oxidative damage in HF are suggestive of a gradual form of ferroptotic-like cell death, which is consistent with the relatively long life span of patients and animal models. Ongoing stress from unproductive and compensating cellular processes would likely be an important component. Protein aggregate formation from ferritin or other proteins may cause other forms of cumulative damage indirectly contributing to cell death on a time scale differing from ferroptosis, which is rapid. Further complicating the issue is that normal brain iron concentration increases with age (Weinreb et al., 2012) paralleling the likelihood of onset of several different neurodegenerative diseases, as is found with HF, and most of these diseases are suggested to have a mechanism-based iron component to their etiology.

## The Role of Ferroptosis in HF—Similarities and Differences

Drawing a complete analogy between cell death from a long-term process like HF and a short term, small molecule-induced death process like ferroptosis is difficult in that the short term process would not necessarily exist long enough to allow production of long term, cumulative cellular damage. Both processes critically involve iron, and they have ROS and phospholipid damage as components. In HF ferritinophagy is likely eliminated or greatly reduced, with iron levels gradually increasing from four-fold pore leakage, which does not occur in ferroptosis. Although the time scales differ, both HF and ferroptosis appear to respond to increasing levels of iron in an increasingly negative manner. Because HF has an overproduction of ferritin and a C-terminus that allows for iron bridging, aggregates and IBs form over the long term. This process apparently does not occur in ferroptosis, but it would be interesting to examine what would occur if ferroptosis could be slowed into a more long-term process and the results compared to other forms of cellular dysfunction in neurodegenerative diseases. Perhaps protein aggregates from ROS damaged proteins and iron would occur in an extended form of ferroptosis more closely paralleling neurodegenerative diseases. In addition, GSH levels fall with age, in ferroptosis, and in neurodegenerative diseases (Emir et al., 2010), again pointing toward a parallel with a more long term ferroptotic-like state. Thus, the designation of long term ferroptotic-like state in HF is meant to capture the dependence on iron, ROS, lipid and protein damage, and stress, and perhaps declining GSH levels in both processes, but not to draw a complete molecular level parallel. Interestingly, this discussion brings up the question of time scales in contributions to neurodegenerative diseases.

## Conclusion

Not long ago, one could attend a seminar on major neurodegenerative diseases and hear no or only passing mention of transition metal ions. The same absence is still true of some current textbooks. These lectures and texts generally focused on aggregation and fibrillization alone. However, the current literature is becoming increasingly clear concerning the very important connections between iron and AD, PD, Prion disease, motor neurone disease, and other chronic slowly-progressing neurodegenerative diseases (Biasiotto et al., 2015; Ndayisaba et al., 2019; Weiland et al., 2019). This review emphasizes the complexity of the cellular problem at hand with respect to interactions of iron with certain proteins and small molecules as caused by HF. The inability of ferritin containing the mutant subunit to properly store iron and its modified and extended C-terminus have several consequences. These are seen as measured observables in elevated iron concentration, overproduction of ferritin, enhanced protein and lipid oxidation, ROS generation, and aggregation and deposition of ferritin into IBs. The elevated iron level in HF is causal to both production of ROS and ferritin overproduction to attempt to compensate for iron elevation. The mutant C-terminus and iron together foster ferritin aggregation leading to IBs. However, these cellular changes are not static endpoints and they themselves have far reaching cellular involvements and consequences.

ROS are generated (1) by reductants and oxygen at iron improperly coordinated by small molecules, mutant ferritin, and ferritin or other proteins that are unfolded or aggregated, (2) secondarily as protein and phospholipid reaction products from primary ROS attack, and (3) from enzymes that miscycle or for defensive purposes. Additionally, improperly coordinated iron may cause interconversions between various ROS, and these may increase toxicity. Once damaged by ROS, proteins tend to unfold exposing their hydrophobic interiors that associate and form aggregates independent of, but also potentially driven further by, iron binding. Additionally, this process may be cyclical because ROS can be generated from iron on aggregates and ROS attack and damage helps aggregate proteins. Cells must expand resource outlays and shift resources away from other tasks to attempt to correct for ROS overproduction, protein and lipid damage, and disposal of aggregates, which are common stressors in neurodegenerative diseases. These cellular outlays can take the form of additional synthesis of proteins to attempt to restore iron homeostasis, destroy ROS, repair proteins and phospholipids, and eliminate and transport protein aggregates, and also to maintain redox balance and energy availability for these as well as other normal cellular processes. These many processes are materially and energetically costly and stress the cell. *Elevated iron and lipid peroxidation products as are found in HF are markers for ferroptosis, which may be occurring at a moderate level as the cells try to cope with the above-mentioned multiple stressors*.

In HF, excess ferritin synthesis, in an attempt to control iron levels, diverts ribosomal proteins and energy away from other necessary cellular synthesis. Ferritin disposal is also a complex, interdependent and a somewhat redundant system perhaps involving both the proteasome and the lysosome, which require synthesis and energy. However, the extent and distribution between these becomes unclear in HF especially with respect to soluble ferritin vs. ferritin aggregates. Elements of the proteasomal machinery are evident in IBs in HF, however such is not likely to effectively clear excess ferritin because of the large size of the 24-mers and aggregates making the lysosomal clearance primary. NCOA4 binding to ferritin is required for ferritin to be transported to the lysosome and undergo ferritinophagy, but inclusion of the mtFTL subunit itself, or the resulting aggregation it induces, could hinder the binding of NCOA4 to ferritin for disposal thus enhancing accumulation into IBs. Under the circumstance that ferritin aggregates are not disposed of by ferritiophagy, the proteasome likely attempts compensation. However, the formation of IBs in HF suggests that the process of IB formation outpaces the ability of both proteasomal and lysosomal degradation, and proteasomal proteins may be synthesized but not effectively used for ferritin clearance. These several cellular processes meant to compensate or correct for the presence of mtFTL are all energetically and materially costly, and stress the cell.

This review makes clear the complex interrelationships in HF as the cells are stressed and muster their efforts at maintaining overall homeostasis. The two key pathogenic mechanisms for the phenotypic expression of the disease are best explained (1) as a loss of normal ferritin function with decreased iron incorporation that triggers intracellular iron accumulation and overproduction of ferritin polypeptides, and (2) a gain of toxic function through ROS production, ferritin aggregation, and oxidized protein and lipid formation (Muhoberac and Vidal, 2013; Vidal and Ghetti, 2015). However, this phenotype does not fully emphasize the layered cellular damage signified by oxidation and aggregate formation, which may have parallels in a number neurodegenerative diseases and lead to a major loss of normal cellular function. The responses to (1) and (2) above tax cells energetically, and with respect to material resource availability and management, causing gradual, but cumulative damage. Even proteasomal action is inhibited by aggregated proteins. Interestingly, it is found that there is only a modest increase in iron levels (10–20%) in cells and animal models vs. the rather large associated increase in ferritin (200–400%) found with them. Moderate iron level increases make sense in that a substantial increase would likely be more rapidly lethal to the cells through ROS production and aggregate formation, and could shut down ferritinophagy completely if any portion was still functional. Both humans with HF and animal models do exhibit moderate life spans and gradually, not rapidly form IBs. Still, because iron is redox active even low concentrations can cause ROS damage, especially if the cellular stress is long term and to some extent cumulative.

The presence of IBs is a diagnostic marker for HF, and they are increased much more than the iron level. The chronic-progressive increase of IB (number and size) through the duration of the disease points toward a serious failure of the systems attempting to manage cellular ferritin disposition, i.e., lysosomal degradation through ferritinophagy. NCOA4-FTH1 binding is inhibited by high iron concentration in purified protein studies and NCOA4 degradation is enhanced (Mancias et al., 2015; Gryzik et al., 2017), however these may not be enough to substantially suppress ferritinophagy. Thus, the modest iron increases found in *in vivo* studies of HF do not rule out an additional, perhaps dominant contribution to hindered NCOA4-ferritin binding and decreased ferritinophagy that is mechanistically separate from the two above-mentioned iron level effects. *Importantly, it is likely that the presence of the partially unfolded mtFTL subunit in ferritin would decrease NCOA4-ferritin binding directly through hindering the NCOA4-FTH1 interaction and also through enhanced ferritin aggregation making NCOA4-FTH1 interaction spatially difficult to achieve*. Additionally, aggregates of ferritin would be difficult to transport to the lysosome or aggresomes. This may provide a mechanism for the gradual buildup of aggregates into dispersed IBs that is not dependent on high iron levels.

Even recently, the contribution of iron-induced aggregation has been suggested to be unimportant in the etiology of HF (Luscieti et al., 2010; Levi and Rovida, 2015). Such aggregation can occur through C-terminal bridging of ferritin containing the mtFTL subunit or by ROS oxidation and proteolysis of ferritin destabilizing its structure. ROS formation originates from a variety of cellular sources with improperly coordinated iron, such as small LIP carrier molecules, the mutant FTL C-terminus, protein proteolysis products, or unfolded or aggregated proteins. Reductants such as ascorbate are clearly available in the brain and the Haber-Weiss and Undenfriend-type reactions use ascorbate and iron to generate various damaging ROS depending on iron coordination. The process of protein aggregation, especially with ferritin containing the mtFTL subunit unraveled C-termini, will sample a variety of conformations as the initial aggregates are formed. *It follows that iron, especially but not necessarily in great excess, could sample a variety of coordinations with some being facile ROS producers, and this sampling would likely occur not only with ferritin and HF but with other proteins and neurodegenerative diseases*. Interestingly, the finding that HF and HF models produce a substantial fraction of SDS-insoluble ferritin brings up the possibility that the IBs, once formed, could hinder penetration of a reductant as large as ascorbate from their interior and keep iron safely six coordinate and oxidized. ROS generation on the surface of the IBs may occur, but the surface area to volume ratio would suggest a net reduction in damage by IB mutant ferritin over what would occur with small dispersed mutant ferritin aggregates and iron. The proteasome may target more than mutant ferritin alone, including repair or removal of other ROS-damaged proteins. However, IB buildup, transport and elimination is another issue.

On the negative side, the size and number of IBs increase with age, and formation of a large number of IBs would likely hinder crucial cytoplasmic transport or nuclear processes. Non-mutant ferritin aggregates are known to associate with tubulin (Hasan et al., 2006) and ferritin associates with kinesin (Jang et al., 2016), suggesting the mutant ferritin aggregates could be problematic in cytoskeletal transport disruption in that transport requires these proteins. Disruption of transport would scale with the age of the cells, and not only hinder ferritinophagy, but a number of other crucial cellular transport processes. Thus, damage could occur in a cyclical manner through inhibition of required ongoing cellular processes involving transport and clearance.

**Figure 6:**
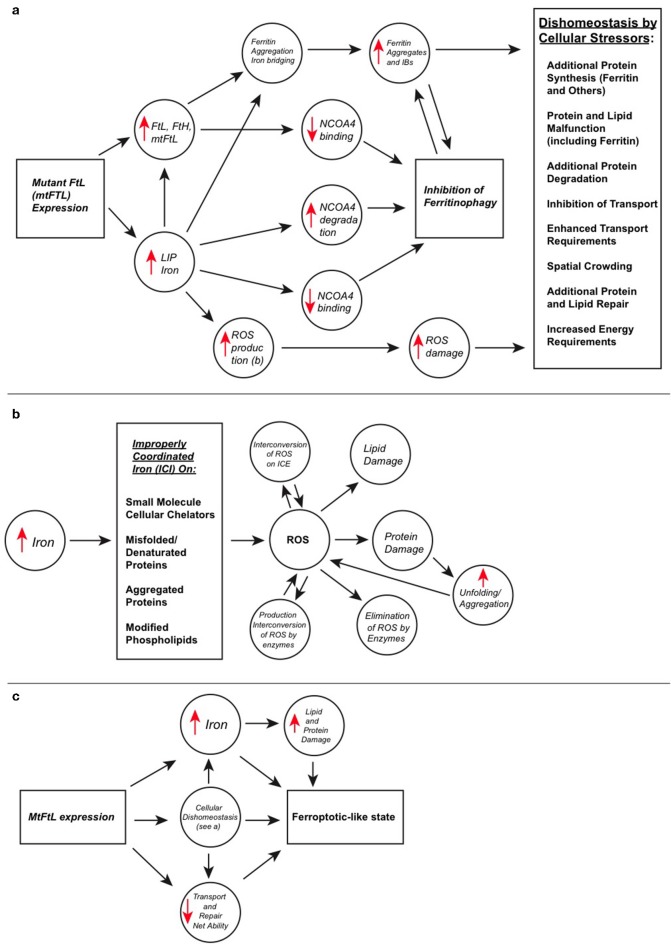
Detailed interrelationships in HF between iron accumulation, ferritin overexpression, ROS generation, other stressors, and a ferroptotic-like cellular state. **(a)** The mutant ferritin subunit (leftmost box) is incorporated into ferritin 24-mers, which form spherical shells with altered four-fold pores and are functionally defective. Mutant-containing ferritin does not incorporate iron appropriately causing increased LIP iron concentration and a feedback loop leading to ferritin overexpression to try to control iron levels. Increasing iron causes ROS formation through multiple paths (see **b**) and cellular damage, as well as enhanced aggregation of mutant-containing ferritin through bridging unraveled C-termini and from ROS damage. There are at least three mechanisms that can inhibit ferritinophagy involving NCOA4 (three vertical circles), two caused by increased iron levels and the third by the C-terminal mutation, in addition to ferritin aggregates directly inhibiting microtubule transport and proteosomal degradation, leading to buildup of ferritin aggregates and IBs. The combination of accumulation of ferritin aggregates and ROS damage causes cellar dishomeostasis leading to several forms of cellular stress (rightmost box). **(b)** ROS can originate with cellular iron that is improperly coordinated (see box), as well as by enzymes that misfire or form them normally. ROS can be converted to other ROS forms, sometimes more toxic, by improperly coordinated iron or native enzymes. Certain enzymes have the function to eliminate ROS as cellular protectors. Both lipid and protein damage occur from ROS leading to local cellular loss of function, and protein damage can lead to aggregation further enhancing and spreading ROS production. **(c)** Mutant-containing ferritin clearly leads to increased iron levels, which in turn causes ROS-induced protein and lipid damage in HF. However, compensating for the long-term dishomeostasis (see box in **a**) caused by mutant ferritin stresses the cell, diverting resources and energy away from bulk metabolism and taxing cellular transport and repair mechanisms, which contributes to a long-term cellular weakening process. Increased iron and lipid peroxidation, which are both found in HF, are hallmarks of ferroptosis, which leads to cell death and is associated with several neurodegenerative diseases. The causative factors in cell death in HF appear to be not only iron accumulation and ferritin aggregate formation, but additionally, a combination of several gradual but cumulative forms of overall stress (**a**, rightmost box) causing cellular compensatory breakdown that taken together cause a long-term ferroptotic-like cellular state.

The overall pathway in HF from mutant ferritin expression to cellular dysfunction and death is a long-term stressful process. The gradual nature of HF development suggest that cells do cope with ferritin overexpression and elevated iron for some time before becoming critically dysfunctional. Increased iron in the LIP leads to ROS production and overproduction of ferritin with elimination of ferritin compromised (Figure 6a). These two misfunctions lead to a more general cellular dishomeostasis by causing several stressors taxing the cells and diverting energy and material away from bulk metabolism, transport, and repair. ROS production is strongly associated with improperly coordinated iron (Figure 6b) which can become more numerous as the ferritin mutant, is aggregates, and other misfolded proteins and their aggregates accumulate and provide iron-binding sites. Cells expend synthesis energy to eliminate ROS and repair damage. The increases in iron and lipid damage, which are known markers for ferroptosis, are clearly observed in HF, but the moderate increase in iron levels and gradual, cumulative nature of the overall damage suggests a more general, long term, cellular dishomeostasis. Still, the moderately elevated iron and ROS lipid damage, modified by the gradual, cumulative cellular dishomeostasis, may produce a long term ferroptotic-like state (Figure 6c). It is worth mentioning a cautionary note that many *in vivo* and *in vitro* studies of iron homeostasis and cellular stress are made (1) under conditions of chelator, reductant, and ROS concentrations and (2) over time scales that are designed to elicit a change that mimics some aspect of a disease state. However, these studies may be unrepresentative of the low physiological concentrations and long time course of the disease under investigation, especially if it takes years to develop such as with most neurodegenerative diseases.

This review on HF details biochemical aspects of metal ion interactions, protein misbehavior, and cellular responses that may have parallels or commonality to other neurodegenerative diseases, especially with respect to ROS formation and damage, as well as protein aggregation, inclusion formation, and disposal pathway inhibition. With respect to HF treatment, we can add two additional approach to our previous suggestion of simultaneous administration of a radical scavenger and appropriate iron ion chelator (Muhoberac and Vidal, 2013). Importantly, *Ftl*^−/−^ mice show that because FTH1 ferritin homopolymers can maintain brain iron homeostasis in a mouse model, there is the potential for development of a therapeutic approach for HF treatment using RNA interference to induce sequence-specific post-transcriptional gene silencing of mutant FTL or alternatively both, wild-type and mutant FTL alleles (Li et al., 2015). In addition, drug-based manipulation or enhancement at some level of the cellular disposal mechanisms may reduce ongoing ferritin and aggregate accumulation, and thus reduce cellular stress enhancing survival. It is hoped that researchers in the field of neurodegeneration will be able to find parallels and apply them to their individual systems of study and lead to more robust treatments.

## Author Contributions

Both authors listed have made a substantial, direct and intellectual contribution to the work, and approved it for publication.

### Conflict of Interest

The authors declare that the research was conducted in the absence of any commercial or financial relationships that could be construed as a potential conflict of interest.
